# Special Knowledge of Flying Conditions Imperative

**Published:** 1918-10-05

**Authors:** 


### THE ROYAL AIR FORCE MEDICAL SERVICE.

### Special Knowledge of Flying Conditions Imperative.

THE phenomenally rapid development of the Air Forces during the war, culminating in the recent amalgamation of their component Services, has created for the Medical Services a problem which urgently demands solution. The ailments of pilots and observers differ so materially from those of all other occupations that specially trained doctors are essentially necessary for their prevention and treatment. The supply of doctors in the open market is now so limited that they cannot be selected from that source; they are mainly transferred or borrowed from the sister Services on expedient principles, they are neither specially selected nor previously trained.

The opinion is strongly maintained in the R.A.F. that their doctors should themselves be personally acquainted with the conditions of flying at all heights, more especially at 18,000 feet, for it is at this height and over that ailments of any importance originate. The R.A.F. doctor must therefore be young and sound, and he must be acquainted with the effects of high altitudes on the circulation and respiration, and of the effects of air warfare on the nervous system, including those of psychic origin. The Medical Research Committee are engaged on air questions, and have already published a number of papers on the subject, and the Air Commissioners have circulated others. The R.A.F. Hospital at Mount Vernon, Hampstead, provides much of the clinical material for research. It is a large establishment, to the staff of which are attached eminent specialists in the subjects connected with flying. The formation of courses of instruction should therefore present no difficulties; doubtless such courses are contemplated, and it is our fervent hope that such intentions may materialise at the earliest possible date, for the need is urgent.

The psychoneuroses of flying should be given a prominent place in the instruction. It is a subject which is not realised by the ordinary practitioner, and one which can be taught by experts only; yet it is more deeply concerned in the war wastage of an Air Force than any other personal factor. After the course of instruction in medical subjects the candidates should be in a condition to appreciate—and to profit by—a thorough course of flying under varying conditions. Air work disorganises the human mechanism in a peculiar manner. Up to 10,000 feet there are no marked disturbances—a mild form of mountain sickness, characterised by headache, pallor, nausea, or vomiting occurs among the susceptible (about 60 per cent.), but at 18,000 feet and over the full effects of high altitudes are manifested in direct proportion to the reserve nerve force possessed by the individuals. The symptoms are a markedly quickened pulse, urgent dyspnœa, headache, vertigo, and, at times, nausea and vomiting; malaise, inordinate thirst, and anorexia are later symptoms, and hæmoptysis occurs occasionally. During the winter, the effects of cold also are experienced; frostbite is not uncommon among the observers.

Ordinarily a pilot on reaching 15,000 feet commences to be deaf, and at 18,000 feet he cannot hear the telephone; in addition he suffers from throbbing temples and apnœa; then, if he is at the Front, his nervous system is subjected to continuous shocks, in addition to the whirr of the engines, the rattle of machine-guns, and the emotional shocks of manœuvre and combat. On descending, the apnœa disappears, but, more especially if the descent is sudden, a complex vertigo, accompanied by heterophoria, is manifested, there is a disturbance of equilibrium and of space orientation, frequently resulting in faulty landing. This is due to a violent excitation of the semicircular canals by variation in pressure and circulatory disturbances, and by reflex influences, ocular and psychic. During voluntary landings this aberration in the perception of space is overcome by circling round the aerodrome.

The nervous effects of flying are progressive and cumulative, insidious and subtle; the flying officer is young, proud, and sensitive, and he struggles to the end in vain endeavour to shake off his vague and inexpressible nervous symptoms; he will not report sick, and it is for this reason that doctors competent by knowledge and experience are required to recognise and to study the prevention of war wastage from these causes. The competent observation of psychological conditions is based on personal confidence and esteem; it is therefore essential that the medical officer should live with his unit, and be exempted from frequent changes; a hint from a squadron leader or flight commander to such an officer of any unusual deviation from the normal among his pilots would constitute a basis for tactful observation and action. The maintenance of mental stability in pilots is governed by rest and change of scene at sufficiently short intervals and for sufficiently long periods. After twenty-five hours' flying, in flights of from two to three hours, usually occupying three weeks, an average flyer requires from three to four days' complete rest and change, and after three months a longer period, from two to three weeks; the most economic periods of leave can be fixed only after full scientific consideration of the conditions. The medical officer would receive valuable aid from a record of flying hours, which should be kept.

It is after the first three months' flying that a careful watch is required. Even after a fortnight's rest a considerable proportion of flyers soon develop psychosthenic symptoms; if symptoms of battle-shock are superadded, a complete change of scene is essential. The sufferer must be removed entirely from the sound of engines or the rattle of machine-guns; light duty at a home aerodrome is not sufficient. Sufficient facts have here been recorded to indicate the imperative necessity for

**The Royal Air Force Medical Service**—(*continued*).

special training for the R.A.F. Medical Service. The Navy and the Army have technical medical colleges at Haslar and in London which have fully justified their existence; may we hope for a similar college for the Air Forces? We understand that a new central hospital is under construction at Salisbury, and the selection of this clinical centre for the purpose in the immediate future presents a favourable opportunity.

